# Six-minute rowing test: a valid and reliable method for assessing power output in amateur male rowers

**DOI:** 10.7717/peerj.14060

**Published:** 2022-09-22

**Authors:** Álvaro Huerta Ojeda, Miguel Riquelme Guerra, Walter Coronado Román, María-Mercedes Yeomans-Cabrera, Rodrigo Fuentes-Kloss

**Affiliations:** 1 Núcleo de Investigación en Salud, Actividad Física y Deporte ISAFYD, Universidad de Las Americas, Viña del Mar, Chile; 2 Magíster Medicina y Ciencias del Deporte, Facultad de Ciencias, Universidad Mayor, Santiago, Chile; 3 Facultad de Educación Física y Deporte, Escuela Naval “Arturo Prat”, Valparaíso, Chile; 4 Facultad de Educación, Universidad de Las Americas, Viña del Mar, Chile; 5 Departamento de Ciencias de la Salud, Facultad de Medicina, Pontificia Universidad Católica de Chile, Santiago, Chile

**Keywords:** Rowing, Amateur rowers, Power output, Validity, Reliability

## Abstract

**Background:**

Standardized tests are currently available to assess power output in elite rowers. However, there are no valid and reliable tests to assess power output in amateur rowers.

**Objective:**

This study aimed to determine the validity and reliability of a 6-min rowing ergometer test (6-min_RT_) as a predictor of power output (PO) in amateur male rowers.

**Methods:**

Twelve male amateur rowers were part of the study. All participants were instructed to perform an incremental test (IT), a 6-min_RT_ test, and a retest. The validity of the 6-min_RT_ was determined by comparing maximum oxygen uptake (VO_2max_) and power output (PO) between the IT and 6-min_RT_. Reliability included the coefficient of variation (CV), intra-class correlation coefficient (ICC), and mean standard error between the 6-min_RT_ test and retest. The significance level was *p* < 0.05.

**Results:**

There was no significant difference in VO_2max_ in both IT and 6-min_RT_ (*p* = 0.18), while the mean power output (P_mean_) generated in the 6-min_RT_ equaled 91.96% of the maximal aerobic power (MAP) generated in the IT (*p* = 0.004). Reliability analysis for the 6-min_RT_ showed a CV = 0.50% and ICC = 0.97 for distance, a CV = 1.85% and ICC = 0.96 for P_mean_.

**Conclusion:**

From a ventilatory and mechanical point of view, the 6-min_RT_ is a maximally valid test for establishing MAP in amateur rowers. Also, the 6-min_RT_ evidences a high degree of agreement between days. Therefore, the 6-min_RT_ is a valid and reliable test for assessing PO in amateur male rowers.

## Introduction

Several factors determine optimal performance in competitive rowing (Huntsman, Drury & Miller, 2011; Muniesa & Díaz, 2010; Rich, Pottratz & Leaf, 2020). Among them were high fitness and technical development (Huntsman, Drury & Miller, 2011), good boat stabilization (Muniesa & Díaz, 2010), and correct synchronization between all the boat members (Rich, Pottratz & Leaf, 2020). Furthermore, as measured on water, the performance depends on external factors, including environmental conditions (Ingham et al., 2002). Also, considering that in competitive rowing, the contribution of aerobic metabolism ranges between 70–88% and the contribution of anaerobic metabolism fluctuates between 12–30% (Hagerman et al., 1978; Pripstein et al., 1999; Secher, 1993), most research has focused on the aerobic component, mainly maximum oxygen uptake (VO_2max_) (Das et al., 2019; Klusiewicz et al., 2016). For example, elite rowers can exceed 6 LO_2_·min^−1^ in a maximum intensity test (Hagerman et al., 1978). Similarly, it has been observed that elite rowers reach VO_2max_ between 330–360 s (Secher, 1993). Indeed, this high aerobic level and other conditioning components allow elite rowers to go under 360 s in an individual test—the 2,000 m time trial (2,000m_TT_) performance (Silva, 2016).

Currently, the most widely used physical test to determine sports performance in rowers is the 2,000m_TT_ (Silva, 2016; Turnes et al., 2020). The reason for using the 2,000m_TT_ is that the advent of rowing ergometers has facilitated training and provided a controllable and repeatable tool to assess rowing performance (Ingham et al., 2002). Moreover, the usefulness of 2,000m_TT_ as an assessment of rowing performance increases if the gas analysis has been used (gold standard) (Wagner, 1996). In parallel, another variable that conditions rowing performance is the power output (PO) (Bourdin et al., 2017; Bourdin, Messonnier & Lacour, 2004). The PO on the rowing ergometer is the result between the stroke rate and the force exerted by the rower in each stroke. Indeed, maximal power output (P_max_) presents the advantage of being obtained with a simple ergometer test without biological measurements (Bourdin et al., 2017). Likewise, the most commonly used test to determine PO in rowers is the 2,000m_TT_ (Silva, 2016; Turnes et al., 2020). In this test, PO corresponding to VO_2max_ (PO at VO_2max_) has been evaluated over 400 watts (W) in elite rowers, surpassing 450 W in heavyweight rowers (Bourdin, Messonnier & Lacour, 2004).

Both PO and VO_2max_ have shown a high correlation in the 2,000m_TT_ in high-level rowers (*r*^2^ = 0.88, *p* < 0.01) (Turnes et al., 2020). However, this correlation decreases when the rowers lever is lower (VO_2max_ *vs* 2,000m_TT_: *r* = −0.55, *p* < 0.012; PO *vs* 2,000m_TT_: *r* = −0.63, *p* < 0.004) (Kendall et al., 2011). In this sense, Klusiewicz et al. (2016) showed a high total error rate when the athletes’ capacity was lower. Likewise, those rowers who take around 360 s to complete the 2,000m_TT_ (Bourdin, Messonnier & Lacour, 2004) can perform this test at a mean power output (P_mean_) higher and closer to VO_2max_ during the entire test. On the other hand, those who take more than 420 s to complete the 2,000m_TT_ must perform this test at submaximal intensities, which is reflected in lower P_mean_ values (Turnes et al., 2020; Bourdin et al., 2017; Huerta Ojeda et al., 2022). Indeed, in a recent study developed by our research group, it was evidenced that during the 2,000m_TT_, amateur rowers reach a P_mean_ of 276.2 ± 23.9 W and 269.5 ± 31.3 W at VO_2max_. These values correspond to 80.5% and 82.6% of the maximal aerobic power (MAP) evaluated in that Incremental Test (IT), respectively (Huerta Ojeda et al., 2022). If the training loads of amateur rowers are based on the P_mean_ obtained in the 2,000m_TT_ (without gas analysis) (Wagner, 1996), this 20 percentage point difference between the IT and the 2,000m_TT_ could lead to inaccuracies. In the latter case, amateur rowers should explore a test that allows evaluating, in the field, a close P_mean_ to VO_2max_, without the presence of fatigue in the last part of the test (6-min rowing test (6-min_RT_)) (Huerta Ojeda et al., 2022).

As evident, ventilatory and mechanical parameters play a predominant role in rowers of all categories (Hagerman et al., 1978; Wagner, 1996). Likewise, it was proven that the 2,000m_TT_ is an accurate test to determine PO in elite rowers, demonstrating a high concordance with VO_2max_ (Turnes et al., 2020). However, the lower fitness level of amateur rowers causes a low concordance between the 2,000m_TT_ with VO_2max_, P_max_, and P_mean_, generating low reliability for this category (Huerta Ojeda et al., 2022). Added to the above, due to the high cost of laboratory tests (gas analysis) (Wagner, 1996), most of the training performed by amateur athletes is based on the results of indirect field tests (Metaxas et al., 2005). Despite this evidence, and considering that amateur rowers must complete the 2,000m_TT_ at submaximal intensities—a farther away P_mean_ from VO_2max_ than elite rowers—this test is still used to evaluate this category, generating impressions in quantifying training loads (Turnes et al., 2020; Bourdin et al., 2017; Huerta Ojeda et al., 2022). Therefore, amateur rowers lack a valid and reliable test, following this category’s aptitude and technical development, for determining PO on the field. Consequently, the main objective of this study was to determine the validity and reliability of the 6-min_RT_ as a predictor of MAP in amateur male rowers.

## Materials and Methods

### Participants

Twelve male amateur rowers from the Naval Academy “Arturo Prat” participated voluntarily in this study. The main inclusion criterion was the VO_2max_ assessed in the initial test (IT). This initial evaluation made verifying the fitness level of the male amateur rowers possible. In this regard, all participants in the study presented a VO_2max_ ≤ 65 mLO_2_·kg^−1^·min^−1^. Also, it was found that the rowers trained less than 6 h per week. All these antecedents allowed defining the participants as amateur rowers. In contrast, those who could not perform the IT and the 6-min_RT_ correctly were eliminated during the study. Statistical software (G*Power, v3.1.9.7; Heinrich-Heine-Universität, Düsseldorf, Germany) was used to calculate the sample (Faul, 2020). The combination of tests used in the statistical software to calculate the sample size was as follows: (a) t-test, (b) Linear bivariate regression: One group, size of the slope, and (c) *A priori*: Compute required size – given α, power, and effect size. Tests considered two tails, slope H1 = 0.67, α-error < 0.05 and a desired power (1-β error) = 0.8, slope H0 = 0.00, standard deviation (SD) σ_x = 0, and SD σ_y = 0, the total sample size was 12 participants. All participants were informed of the study’s objective and the possible risks of the experiment. Before applying the protocols, all amateur rowers signed the informed consent form in person. The study and the informed consent were approved by the Scientific Ethical Committee of the Universidad Mayor, Santiago, Chile (registration number: 197_2020) and developed under the ethical standards for exercise and sports sciences (Harriss, Macsween & Atkinson, 2019).

### Design

A repeated measures design was used to compare test-retest inter-day reliability for different PO, speed, VO_2_, and Heart Rate (HR) variables collected during the 6-min_RT_ exercises. All study participants attended the laboratory for 3 days at 72-h intervals. Also, participants did not exercise between assessment days. This time between races ensured the physical recovery of the rowers.

**Figure 1:**
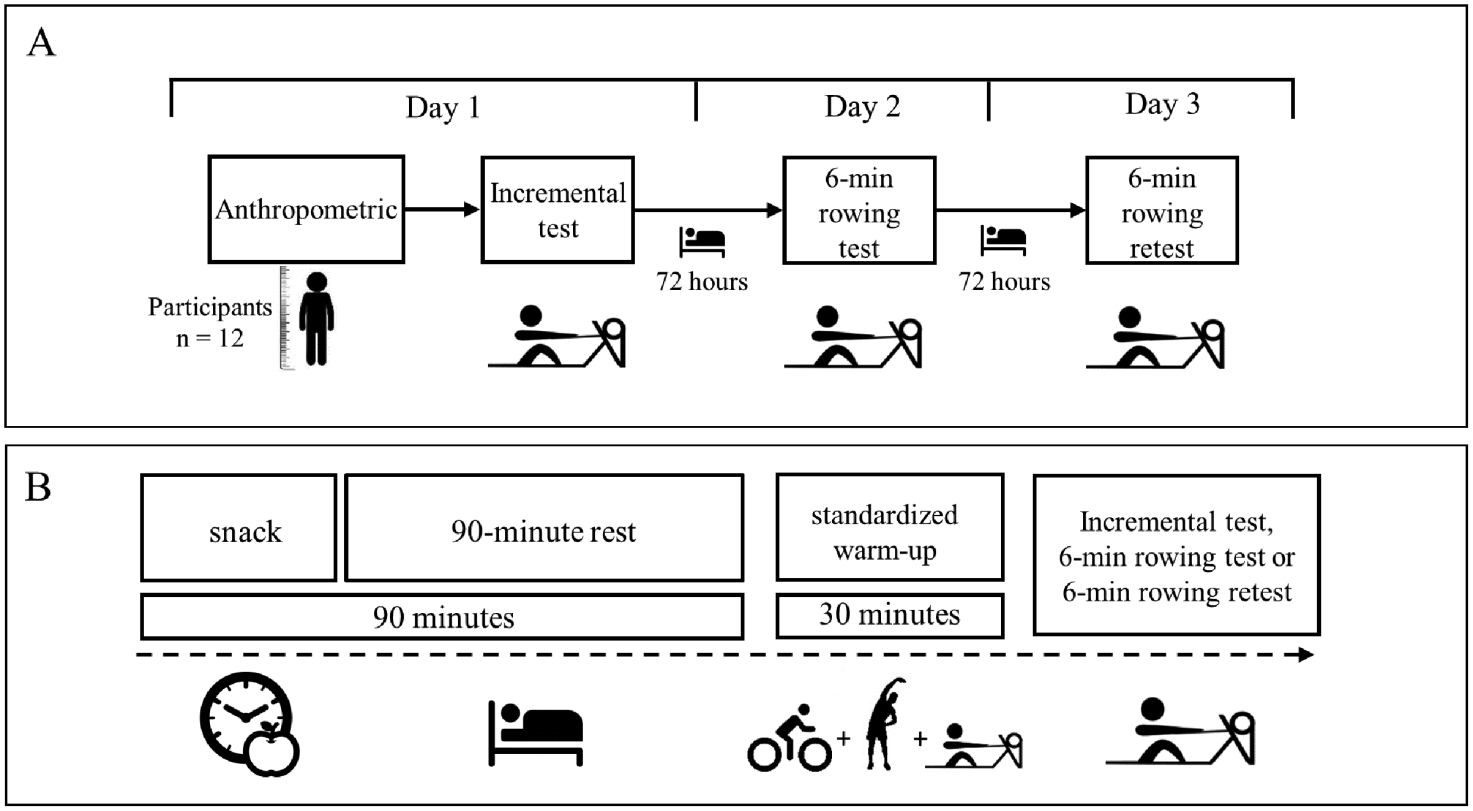
Research design.

During the first visit, basic anthropometric assessments and the IT were performed. The 6-min_RT_ test and retest were performed on the second and third, respectively. All the tests were performed with a rowing machine (Concept2 Model D, monitor PM5, Morrisville, VT, USA) using a drag factor of 111–114 (Fig. 1A).

### Anthropometric measurements

For the characterization of the sample, weight, height, body mass index (BMI), and body fat percentage were evaluated. These evaluations were performed on the day, 30 min after snack intake. This ensured that the participants were evaluated in a euhydrated condition. The body fat percentage was assessed using an impedance meter (Tanita Inner Scan, BC-554® digital scale, Tokyo, Japan).

### Snack

The snack is intended to prevent athletes from starting evaluations with a low blood glucose level (Ojeda et al., 2019). It was a carbohydrate load before the IT and 6-min_RT_ test and retest. All participants were available 2 h before the tests in a fasting condition. The snack consisted of 2 g of rapidly absorbed carbohydrates per kg of body weight (Fig. 1B).

### Standardized warm-up

The warm-up consisted of 10 min on a bicycle (Airbike Xebex® resistance, ABMG-3, USA). The warm-up intensity was between 60–70% of the theoretical maximum HR, calculated through the formula: 208-(0.7 * age) (Tanaka, Monahan & Seals, 2001). Five minutes of upper and lower extremity ballistic movements were then included. After, athletes rowed for 5 min between 60–70% of the theoretical maximum HR (Tanaka, Monahan & Seals, 2001), and finally, there was a 10-min rest (this time was used to install the mask and HR sensors) (Fig. 1B).

### Oxygen uptake

VO_2_ and other ventilatory parameters were evaluated for all tests with an automatic gas analyzer system (model Quark CPET; Cosmed, Rome, Italy). The analyzer was calibrated strictly according to the manufacturer’s recommendations before testing. The data were processed through a laptop computer that calculated the results using software developed by the manufacturer. All ventilatory parameters were averaged at 15 s intervals. Absolute and relative VO_2max_ (VO_2max_ abs and VO_2max_ rel, respectively) and other ventilatory parameters at VO_2max_ (pulmonary ventilation at VO_2max_ (VE at VO_2max_) and respiratory exchange ratio (RER) at VO_2max_ (RER at VO_2max_)) were considered when VO_2_ kinetics generated a variation of less than 150 mLO_2_·min^−1^ between 15 s intervals (Howley, Bassett & Welch, 1995).

### Incremental test

This test aims to progressively reach the highest oxygen consumption with an equivalent PO or MAP. During the test, the participants received verbal encouragement from the research team (Neto et al., 2015). VO_2max_, RER, VE, and carbon dioxide production/oxygen consumption (VCO_2_/VO_2_) was performed with an IT in a rower-ergometer. Each step lasted 1 min in this IT, starting with 100 watts (W) and increasing 50 W in each step until exhaustion or the impossibility of maintaining the requested power (Mekhdieva, Zakharova & Timokhina, 2019). The IT was performed on a rowing machine (Concept2 Model D, monitor PM5; Concept2, Morrisville, VT, USA) using a drag factor of 111–114. The latter intensity was also considered as the MAP of each subject. Power data were recorded in the ErgData V1.4.4® app, USA, and downloaded from an account created at https://log.concept2.com/.

### 6-minute rowing test

This test aims to row for 6 min on a rowing machine and achieve the greatest possible distance. The final distance of the 6-min_RT_ is recorded in meters (m). During the test, the participants received verbal encouragement from the research team (Neto et al., 2015). In this way, it was sought that the participants would achieve the greatest possible distance during the study in both the test and retest. The variables evaluated in this test are described in the following sections. This test aimed to generate a new tool for assessing and programming training loads according to fitness and technical development in amateur rowers.

### Power output

The evaluation of the PO for all the tests was performed with a rowing machine (Concept2 Model D, monitor PM5, Morrisville, VT, USA) using a drag factor of 111–114. The PO was recorded stroke to stroke; however, for analysis and synchronization with the VO_2_ y HR, the PO was averaged at 15-s intervals. Subsequently, the P_mean_ for both IT and 6-min_RT_ in test and retest was determined. Also, the PO at VO_2max_ was observed. The power data were recorded in the application developed by the manufacturer (ErgData, V1.4.4® app; Concept2 Morrisville, VT, USA) and downloaded from an account created at https://log.concept2.com/.

### Heart rate

In the IT and 6-min_RT_ in test and retest, HR was evaluated with a heart rate monitor (model H10®; Polar, Kempele, Finland). This device was synchronized *via* Bluetooth with the rowing machine. HR was recorded by rowing stroke by stroke. However, for analysis and synchronization with VO_2_ and PO, HR was averaged at 15 s intervals. Subsequently, the HR mean (HR_mean_) was determined. Also, HR at VO_2max_ was observed. The HR data were recorded in the application developed by the manufacturer (ErgData, V1.4.4® app, Morrisville, VT, USA) and downloaded from an account created at https://log.concept2.com/.

### Data analysis

For all the tests, ventilatory, mechanical, and HR parameters were sorted on a spreadsheet designed for the study. Descriptive data are presented as means and standard deviation (SD). The Shapiro-Wilk test confirmed the normal distribution of the data (*p* > 0.05). The validity of the 6-min_RT_ was determined by comparing ventilatory, mechanical, and HR parameters between the IT and 6-min_RT_. Reliability of the 6-min_RT_ was assessed through the coefficient of variation (CV), intra-class correlation coefficient (ICC), standard error of the mean (SEM), and corresponding 95% confidence interval between the 6-min_RT_ test and retest. Acceptable reliability was determined as a CV < 10% and ICC > 0.85 (Cormack et al., 2008). The criteria for interpreting the strength of the *r* coefficients were as follows: trivial (0.00–0.09), small (0.10–0.29), moderate (0.30–0.49), large (0.50–0.69), very large (0.70–0.89), almost perfect (0.90–0.99) and perfect (1.00) (Hopkins et al., 2009). The t-tests for related samples and the Bland-Altman technique were used to assess concordance between the 6-min_RT_ test and retest (Hopkins et al., 2009). Reliability assessments were performed using a customized spreadsheet (Hopkins, 2000). All other statistical analyses were performed with Prism version 7.00 for Windows® software. The significance level for all statistical analyses was *p* < 0.05.

## Results

The 12 male amateur rowers had the following characteristics: age: 20.3 ± 1.56 years (range: 19–24), weight: 77.5 ± 5.74 kg (range: 66.8–86.2), height: 176 ± 6.80 cm (range: 169–188), BMI: 25.0 ± 2.14 kg/m^2^ (range: 20.5–27.5), body fat percentage: 12.1 ± 3.37% (range: 6.6–18.0), VO_2max_: 52.91 ± 3.38 mLO_2_·kg^−1^·min^−1^ (range: 49.36–60.86).

The first analysis shows that the amateur rowers in the present study reached, during IT, a VO_2max_ equivalent to 4,090.9 ± 265.7 mLO_2_·min^−1^ (range: 3,412.1–4,362.9), a MAP equivalent to 325.5 ± 39.8 W (range: 229.6–375.2), and an HR at finish equivalent to 196.5 ± 6.39 (range: 185.0–202.3). Similarly, in the 6-min_RT_, amateur rowers reached a VO_2max_ equivalent to 3,923.0 ± 363.5 mLO_2_·min^−1^ (range: 3,305.7–4,437.0), a P_mean_ equivalent to 289.8 ± 20.9 W (range: 243.1–324.5), and an HR at finish equivalent to 194.6 ± 5.58 (range: 181.4–198.3). When comparing VO_2max_ values between the IT and 6-min_RT_, both absolute and relative, no significant differences were observed (*p* = 0.16 and *p* = 0.18, respectively). When comparing the MAP reached in the IT, and the P_mean_ reached in the 6-min_RT_, a significant difference was observed between both tests (*p* < 0.004). However, only a CV equivalent to 8.04% was obtained between both tests (the P_mean_ generated in the 6-min_RT_ equivalent to the 91.96% of the MAP generated in IT). When analyzing the HR at finish during the performance of both tests, it is observed that the IT was developed with higher HR values, showing significant differences between both tests (*p* = 0.032) (Table 1).

**Table 1:** Validity of the 6-min rowing test (*n* = 12).

Variables	IT (mean ± SD)	6-min_RT_ (mean ± SD)	*p*	$\Delta$ (95% CI)	SEM (95% CI)	CV (95% CI)	ICC (95% CI)
VO_2max_ mLO_2_·min^−1^	4090.7 ± 265.6	3923.0 ± 363.5	0.16	−167.75 [−416.99 to 81.49]	277.38 [196.50–470.96]	6.92 [4.90–11.75]	0.27 [−0.34 to 0.71]
VO_2max_ mLO_2_·kg^−1^·min^−1^	52.91 ± 3.38	50.85 ± 5.37	0.18	−2.06 [−5.27 to 1.14]	3.57 [2.53–6.05]	6.87 [4.87–11.67]	0.40 [−0.19 to 0.78]
MAP (IT) *vs* PO at VO_2max_ (6-min_RT_) W	325.54 ± 39.82	278.13 ± 18.63	0.0001	−47.42 [−65.90 to −28.94]	20.57 [14.57–34.92]	6.81 [4.83–11.57]	0.61 [0.08–0.87]
MAP (IT) *vs* P_mean_ (6-min_RT_) W	325.54 ± 39.82	289.83 ± 20.91	0.004	−35.71 [−57.92 to −13.49]	24.72 [17.51–41.98]	8.04 [5.69–13.64]	0.43 [−0.16 to 0.80]
HR at finish bpm	196.58 ± 6.39	194.67 ± 5.58	0.032	−1.92 [−3.64 to −0.19]	1.92 [1.36–3.26]	0.98 [0.69–1.66]	0.92 [−0.74 to 0.98]
VE at VO_2max_ L·min^−1^	166.09 ± 22.89	154.13 ± 16.24	0.047	−11.97 [−23.80 to −0.14]	13.16 [9.33–22.35]	8.22 [5.82–13.96]	0.60 [−0.07 to 0.87]
RER at VO_2max_ VCO_2_/VO_2_	1.28 ± 0.07	1.24 ± 0.06	0.045	−0.05 [−0.09 to 0.00]	0.05 [0.04–0.09]	4.02 [2.85–6.82]	0.40 [−0.19 to 0.87]

**Note:**

bpm, beat per minute; CI, confidence interval; HR, heart rate; ICC, intra-class correlation coefficient; IT, incremental test; CV, coefficient of variation; L·min^−1^, liters per minute; MAP, maximum aerobic power; mLO_2_·min^−1^, milliliters of oxygen per minute; mLO_2_·kg^−1^·min^−1^, milliliters of oxygen per kilogram per minute; P_mean_, mean power output; PO, power output; RER, respiratory exchange ratio; SD, standard deviation; SEM, standard error of the mean; VE, minute ventilation; VCO_2_/VO_2_, carbon dioxide production/maximal oxygen uptake; VO_2max_, maximal oxygen uptake; W, watts; 6-min_RT_, 6-minute rowing test; $\Delta$, variation delta; *p*, alpha value.

When assessing the concordance between the 6-min_RT_ test and retest, the distance did not show significant differences between the two tests (test: 1,692.5 ± 42.8 m *vs* 1,696.3 ± 46.8 m, *p* = 0.28, CV = 0.50%, ICC = 0.97), P_mean_ showed no significant differences between the two tests (test: 289.7 ± 21.0 W *vs* 290.8 ± 24.1 W, *p* = 0.62, CV = 1.85%, ICC = 0.96), and HR at the finish did not show significant differences between the two tests (test: 194.7 ± 5.6 bpm *vs* 195.8 ± 6.5 bpm, *p* = 0.08, CV = 0.72%, ICC = 0.96) (Table 2).

**Table 2:** Reliability of the 6-min rowing test (*n* = 12).

Variables	Test (mean ± SD)	Retest (mean ± SD)	*p*	$\Delta$ (95% CI)	SEM (95% CI)	CV (95% CI)	ICC (95% CI)
Distance m	1,692.5 ± 42.8	1,696.3 ± 46.8	0.28	3.83 [−3.7 to 11.4]	8.42 [5.9–14.3]	0.50% [0.35–0.84]	0.97 [0.90–0.99]
Intensity m·s^−1^	4.70 ± 0.12	4.71 ± 0.13	0.24	0.01 [−0.01 to 0.03]	0.02 [0.02–0.04]	0.50% [0.35–0.84]	0.97 [0.90–0.99]
P_mean_ W	289.7 ± 21.0	290.8 ± 24.1	0.62	1.10 [−3.72 to 5.91]	5.36 [3.80–9.10]	1.85% [1.31–3.14]	0.96 [0.85–0.99]
HR_mean_ bpm	182.9 ± 4.9	184.7 ± 5.0	0.07	1.72 [−0.18 to 3.62]	2.11 [1.50–3.58]	1.15% [0.81–1.95]	0.85 [0.56–0.95]
HR at finish bpm	194.7 ± 5.6	195.8 ± 6.5	0.08	1.08 [−0.17 to 2.34]	1.40 [0.99–2.27]	0.72% [0.51–1.21]	0.96 [0.86–0.99]

**Note:**

bpm, beat per minute; CI, confidence interval; HR_mean_, mean heart rate; CV, coefficient of variation; ICC, intra-class correlation coefficient; m, meters; P_mean_, mean power output; SD, standard deviation; SEM, standard error of the mean; W, watts; 6-min_RT_: 6-minute rowing test; $\Delta$, variation delta; *p*, alpha value.

**Figure 2:**
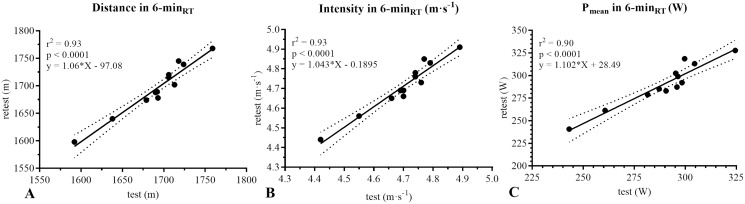
Regression analysis between test and retest of the 6-min_RT_. m, meters; m·s^−1^, meters per seconds; P_mean_, mean power output; W, watts; 6-min_RT_: 6-minute rowing test; *p*, alpha value.

Regression analysis evidenced that the distance (m), intensity (m·s^−1^) and P_mean_ between the 6-min_RT_ test and retest had an almost perfect *r* coefficient (*r*^*2*^ = 0.93, *r*^*2*^ = 0.93 and *r*^*2*^ = 0.90, respectively) (Fig. 2).

**Figure 3:**
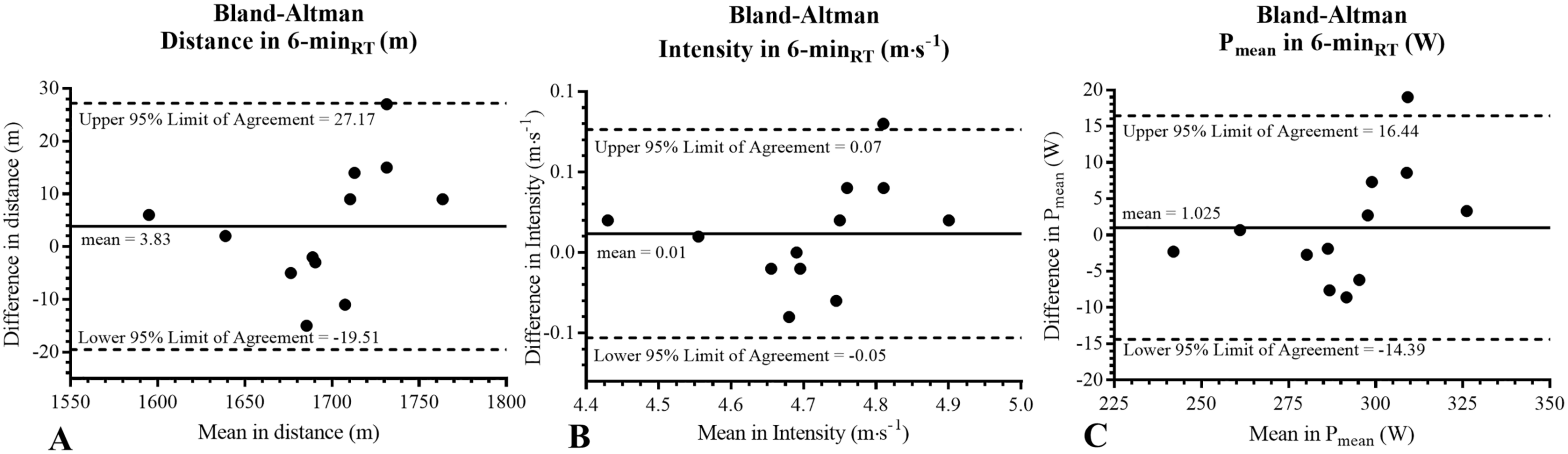
Bland-Altman analysis. The solid line represents the average of the differences between variables evaluated through a 6-min rowing test. The segmented lines represent 95% of the upper and lower confidence limits. m, meters; m·s^−1^, meters per seconds; P_mean_, mean power output; W, watts; 6-min_RT_: 6-minute rowing test; *p*, alpha value.

Upon comparing the mean values and the differences in the distance (m) in the test and retest, Bland-Altman’s analysis showed a common bias of 3.83 ± 11.91 m (95% limits of agreement from −19.51 to 27.17). For the intensity (m·s^−1^), the bias was 0.01 ± 0.03 m·s^−1^ (95% limits of agreement from −0.05 to 0.07), while for the P_mean_ (W), it was 1.025 ± 7.86 W (95% limits of agreement −14.39 to 16.44) (Fig. 3).

## Discussion

This study was designed to determine the validity and reliability of the 6-min_RT_ in amateur rowers. When comparing the VO_2_ between the IT and 6-min_RT_, similar VO_2max_ were observed in both tests (*p* > 0.05). When comparing the MAP (IT) with P_mean_ (6-min_RT_), significant differences were observed between both tests (*p* < 0.05). However, these differences are smaller than those observed between the IT MAP and the 2,000m_TT_ P_mean_ in amateur rowers (Huerta Ojeda et al., 2022). This finding confirms that, from a ventilatory perspective, amateur rowers perform both tests at maximum intensity. Likewise, it was observed that the P_mean_ generated in the 6-min_RT_ is closer to VO_2max_ than the P_mean_ generated in the 2,000m_TT_ (Huerta Ojeda et al., 2022). In parallel, the concordance results for distance, intensity, HR, and P_mean_ between the 6-min_RT_ test and retest show a high reproducibility of this test when applied on different days (Hopkins et al., 2009). These findings suggest that the 6-min_RT_ is valid and reliable for assessing PO in amateur male rowers (Huerta Ojeda et al., 2022).

### Oxygen uptake

Concerning VO_2_ values during the 6-min_RT_ in amateur rowers, our findings showed that the present study participants showed an “excellent” VO_2max_ (52.91 ± 3.38 mLO_2_·kg^−1^·min^−1^) when compared to healthy and physically active individuals (Herdy & Caixeta, 2016). Although these VO_2_ values are qualified as “excellent,” they are lower than those observed in elite rowers (Hagerman et al., 1978; Huntsman et al., 2011). Despite this, the evidence of amateur rowers is scarce (Kendall et al., 2011; Huerta Ojeda et al., 2022). In this sense, Huerta Ojeda et al. (2022) showed that amateur rowers reach 55.9 ± 3.4 mlO_2_·kg^−1^·min^−1^ during the 2,000m_TT_. Specifically, the researchers reported that VO_2max_ was reached 345 s after the start of the 2,000m_TT_, after which time VO_2_ began to decline (Huerta Ojeda et al., 2022), possibly due to low fitness and technical development of the amateur rowers (Huntsman, Drury & Miller, 2011). Likewise, Kendall et al. (2011) showed that, during the 2,000m_TT_, amateur female rowers reached a VO_2max_ equivalent to 2.88 LO_2_·min^−1^, while they took 485.8 ± 10.3 s to complete the test. The same researchers reported an *r* = −0.55 between el VO_2max_ value and time in the 2,000m_TT_ (Kendall et al., 2011). Likewise, there is evidence that the 2,000m_TT_ induces a high blood oxidative stress in rowers with high training status (Kyparos et al., 2009), which allows inferring higher blood oxidative stress in amateur rowers. However, this issue needs further exploration.

Concerning VO_2max_ and rowing performance, there is a big difference between elite and amateur rowers. While elite rowers can complete the 2,000m_TT_ in less than 360 s (Silva, 2016), reaching VO_2max_ (Hagerman et al., 1978; Mahler, Andrea & Andresen, 1984), amateur rowers can only cover 1,696.3 ± 46.8 m in the 6-min_RT_ (360 s). Consequently, the 6-min_RT_ is a test that allows amateur rowers to reach VO_2max_ without experiencing a decrease in this parameter at the end of the race. However, this last issue also requires further exploration.

### Power output

A significantly lower value was observed when comparing the MAP in the IT and P_mean_ performed in the 6-min_RT_ by amateur rowers (*p* = 0.004). However, this CV was only 8.04% between both tests (91.96% of the MAP generated in the TI). Indeed, this is the most relevant background to affirm that shorter tests in amateur rowers (6-min_RT_) allow for measuring P_mean_ close to the PO at VO_2max_ (Huerta Ojeda et al., 2022). On the other hand, the PO kinetics observed in the present study is similar to those described by Mahler, Andrea & Andresen (1984). Specifically, higher PO is observed in the first minutes of the test and a gradual decrease until the last minute. However, it should be noted that the PO generated by amateur rowers is lower than that generated by elite rowers in the 6-min_RT_. The latter far exceeds the 289.8 ± 20.9 generated by amateur rowers in this study (Mahler, Andrea & Andresen, 1984). These differences between categories are conditioned by amateur rowers’ low fitness and technical development compared to higher-level rowers (Huntsman, Drury & Miller, 2011). In this sense, it has been observed that the PO (P_mean_ and P_max_) presents the advantage of being obtained with a simple ergometer test without biological measurements (Bourdin et al., 2017). In this context, based on the results of the present study, the 6-min_RT_ is performed with a P_mean_ close to VO_2max_ and, therefore, allows establishing the MAP in amateur rowers. In fact, it has been shown that those rowers who can maintain a MAP for a longer time have a higher performance; the MAP was the most important predictor of performance in rowers (Turnes et al., 2020). Consequently, the measurement and PO use have an additional advantage since most existing rowing meters provide this information in the field (Bourdin et al., 2017). Only tests according to each rower’s physical and technical level should be applied (in the case of amateur rowers, the 6-min_RT_).

### Reliability of 6-min_RT_

Finally, the results show that the 6-min_RT_ has a high degree of reliability for distance (CV = 0.50%; ICC = 0.97), P_mean_ (CV = 1.85%; ICC = 0.96) and HR_mean_ (CV = 0.85%; ICC = 1.15). In this regard, other reliable tests evaluate physical parameters in rowers (Ingham et al., 2002; Otter et al., 2015; Cheng et al., 2012). However, none of the consulted studies allows the evaluation of P_mean_ in amateur male rowers.

### Limitations

The lack of descriptive information on ventilatory kinetics, PO (P_mean_ and P_max_), and HR in amateur rowers made it challenging to compare our findings. Also, the non-inclusion of women in the study limits the conclusions only to study participants and male amateur rowers. We also believe that future research with amateur rowers should analyze in-depth the kinetics of VO_2_, both the slow and fast components. Finally, future studies should include randomization of participants between tests, thus eliminating any cumulative physiological effects affecting the reliability of the results.

## Conclusions

At the end of the study, it could be observed that the 6-min_RT_ allows amateur rowers to reach VO_2max_. Likewise, the P_mean_ generated during the 6-min_RT_ equals 91.96% of the MAP generated in the IT. For this reason, from a ventilatory and mechanical point of view, the 6-min_RT_ is a maximally valid test for establishing MAP in amateur rowers. Also, the 6-min_RT_ evidences a high degree of agreement between days. Therefore, the 6-min_RT_ is a valid and reliable test for assessing PO in amateur male rowers.

## Additional Information and Declarations

### Data Availability

The following information was supplied regarding data availability: The raw data is available at figshare: Huerta, Álvaro (2022): Dataset. figshare. Dataset. https://doi.org/10.6084/m9.figshare.18092729.v2.
